# *FGFR3* gene alteration and protein expression in upper urinary tract carcinoma: unsuitability of ureteroscopic biopsy specimens for *FGFR3* RNA testing

**DOI:** 10.37349/etat.2026.1002385

**Published:** 2026-07-28

**Authors:** Makito Miyake, Yuki Oda, Nobutaka Nishimura, Sayuri Ohnishi, Kazuki Miyazaki, Takuto Shimizu, Takuya Owari, Kota Iida, Kiyohide Fujimoto

**Affiliations:** IRCCS Istituto Romagnolo per lo Studio dei Tumori (IRST) “Dino Amadori”, Italy; Department of Urology, Nara Medical University, Kashihara 634-8522, Japan

**Keywords:** urothelial carcinoma, gene alteration, FGFR3, RNA, upper urinary tract carcinoma, immunohistochemical staining, biomarker

## Abstract

We aimed to evaluate the suitability of archival ureteroscopic (URS) biopsy specimens for fibroblast growth factor receptor 3 (*FGFR3*) biomarker analysis in upper urinary tract urothelial carcinoma. Thirty-six patients with upper urinary tract urothelial carcinoma who underwent diagnostic URS and radical nephroureterectomy (RNU) were included. Among the 36 RNU-derived tissue specimens, 33 (92%) yielded valid *FGFR3* RNA test results; the remaining three (8.3%) showed test failure because of undetectable amplification by reverse transcriptase-polymerase chain reaction. Of the 33 RNU specimens with valid amplification, eight (24%) had *FGFR3* alterations. Of these eight specimens, two (25%) were valid for *FGFR3* RNA testing; one showed positive concordance with the paired RNU specimen, whereas the other did not. The remaining six specimens (75%) showed test failure. Correlation analysis of FGFR3 immunohistochemical staining scores demonstrated a moderate positive correlation between RNU and paired URS specimens (Spearman’s ρ = 0.47, *P* = 0.004). Our findings indicate that URS biopsy specimens are unsuitable for *FGFR3* RNA testing but may be useful for protein-based biomarker analysis when RNU specimens are unavailable.

## Introduction

The treatment landscape for locally advanced or metastatic urothelial carcinoma (la/mUC) has evolved substantially with the introduction of immune checkpoint inhibitors and antibody-drug conjugates (ADCs) [1]. Based on the positive results of THOR cohort 1 [2], an oral, selective pan-fibroblast growth factor receptor (FGFR) tyrosine kinase inhibitor, erdafitinib (Balversa^®^, Janssen Biotech) [2–4], received full approval from the U.S. Food and Drug Administration in January 2024, European Medicines Agency in August 2024, and Pharmaceuticals and Medical Devices Agency in Japan in December 2024. The identification of alterations in the gene encoding FGFR3 (*FGFR3*alt) in patients with urothelial carcinoma (UC) may facilitate biomarker-guided therapy. Therefore, it is vital to understand the factors affecting FGFR testing results and availability of tissue specimens to guide testing strategies for *FGFR3*alt in patients with la/mUC.

THOR cohorts 1 and 2 are global phase 3 trials comparing erdafitinib with chemotherapy in patients with la/mUC harboring susceptible *FGFR3/2*alt who experienced disease progression after previous treatments [2, 4]. Of the 8,396 available tumor samples from 8,733 patients, 7,293 (86.9%) had valid laboratory test results, whereas the remaining 1,103 (13.1%) showed test failure [2]. A large-scale global ANNAR biomarker study investigated potential factors affecting *FGFR* test results and concluded that adequate tumor tissue quantity, RNA quality, and short archival sample age are critical to ensuring valid FGFR test results [5]. Archival samples collected from primary tumors are preferred over metastatic site samples, regardless of location (upper or lower urinary tract), for *FGFR* testing because of the limited tumor content typically present in metastatic samples. However, in clinical practice, ureteroscopic (URS) punch biopsy specimens collected from primary upper urinary tract UC (UTUC) tumors are often small and limited in quantity compared with transurethral resection (TUR) specimens from primary bladder tumors and radical nephroureterectomy (RNU) specimens. Evidence regarding the performance of URS biopsy specimens remains extremely limited. Importantly, no prior study has quantified RNA failure rates or evaluated the concordance between RNA-based *FGFR3* alterations and FGFR3 protein expression in paired URS and radical RNU specimens.

To address this gap, our study provides a systematic assessment of RNA test validity and immunohistochemical (IHC)-RNA concordance using paired URS/RNU samples from patients with UTUC. This approach allows us to offer practice-informing, incremental insights into the feasibility of URS biopsy specimens for FGFR3 biomarker testing.

## Methods

This retrospective, single-center study was approved by the Nara Medical University Ethics Committee (reference protocol ID: 2891) and conducted in accordance with the principles of the Declaration of Helsinki. Informed consent was obtained from all participants through posters and/or websites using the opt-out method. The medical charts of patients with UTUC diagnosed between 2018 and 2024 were reviewed to collect patient data, including age, sex, T category, and tumor grade. Among these, 36 patients met all the inclusion criteria: (i) pathologically proven UC arising from the renal pelvis and/or ureter; (ii) a history of diagnostic URS examination followed by RNU; (iii) UC cells detected by the standard diagnostic process [hematoxylin and eosin (HE) staining] in both RNU specimens and paired URS biopsy specimens; and (iv) available formalin-fixed, paraffin-embedded (FFPE) tissue specimens from both RNU specimens and paired URS biopsy specimens. All HE-stained slides were independently reassessed by an experienced uropathologist, with clinical staging according to the 8th edition of the Union for International Cancer Control TNM classification [6].

Five-µm-thick unstained sections (*n* = 10) from FFPE tissue were subjected to the RNeasy DSP FFPE kit (Qiagen) for RNA extraction; the Qiagen therascreen^®^ FGFR Rotor-Gene Q reverse-transcriptase-polymerase chain reaction (PCR) assay was performed by SRL, Inc. (Tokyo, Japan) for research purposes. This is a proprietary companion diagnostic assay, and the manufacturer does not disclose primer sequences or housekeeping gene(s). The assay was performed strictly according to the manufacturer’s validated protocol. The *FGFR3*alt assay targets four types of *FGFR3* missense mutations (R248C, S249C, G370C, and Y373C) and two types of *FGFR3* gene fusions (FGFR3::TACC3v1 and FGFR3::TACC3v3). The test results were reported as negative or positive for *FGFR3*alt, the type of alteration if positive, or test failure caused by amplification undetected by PCR.

IHC staining was performed using FFPE tissue blocks as previously described [7]. Mouse monoclonal anti-human FGFR3 antibody (clone B-9; cat. sc-13121; Santa Cruz Biotechnology, Dallas, TX) at a 1:50 dilution was used as the primary antibody. Tumor expression of FGFR3 protein was evaluated in at least three to five independent high-power microscopic fields (400×; 0.0625 mm^2^) of UC cells, which were determined on the basis of cell morphology and tumor architecture. The percentage of positively stained UC cells relative to the total number of UC cells was calculated (1–100%). The intensity of tumor FGFR3 expression (score of zero to three) was determined using the histochemical scoring method (H-score), which was calculated as the product of the staining intensity and percentage of cells (0–100%) stained at a given intensity [7]. The IHC staining results were evaluated by two investigators (Y. Oda and K. Miyazaki) who were blinded to the clinicopathological data.

Continuous variables are expressed as the mean ± standard deviation and compared using the Mann-Whitney U test. Data are presented as scatter plots and boxplots. Categorical variables were compared using the chi-square test or Fisher’s exact test, as appropriate. Correlations of FGFR3 H-scores between paired RNU and URS biopsy specimens were examined using Spearman’s correlation coefficient (ρ value) and linear regression analysis (slope). Absolute values of Spearman’s ρ of < 0.2–0.4, 0.4–0.7, and > 0.7 were considered to indicate weak, moderate, and strong correlations, respectively. PRISM software version 10 (GraphPad Software Inc., San Diego, CA, USA) was used for statistical analyses and data plotting. A *P* value < 0.05 was considered statistically significant.

## Results

**Figure 1:**
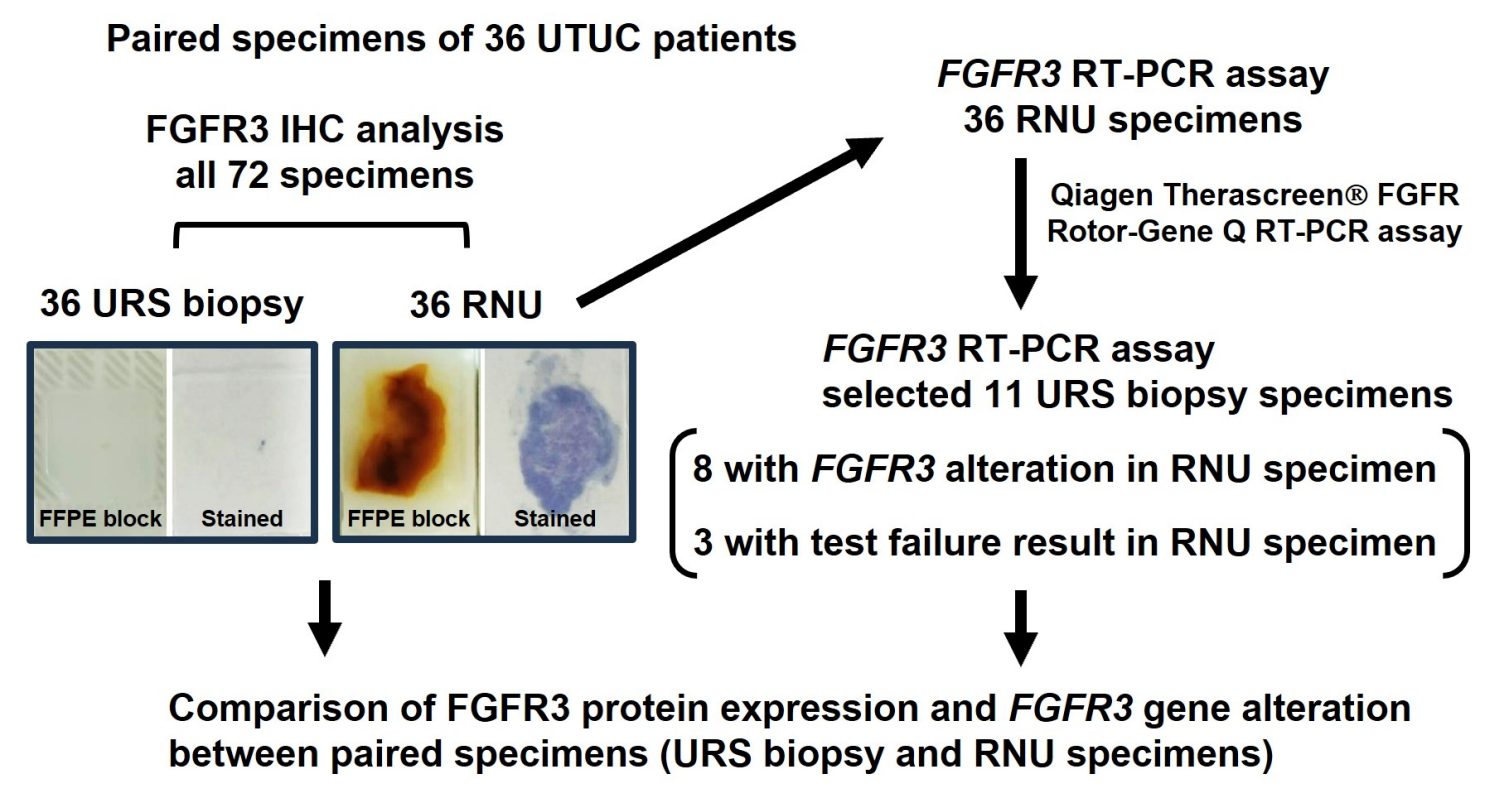
**Flowchart of the study.** FFPE: formalin-fixed, paraffin-embedded; FGFR3: fibroblast growth factor receptor 3; IHC: immunohistochemical; RNU: radical nephroureterectomy; URS: ureteroscopic; UTUC: upper urinary tract urothelial carcinoma.

The characteristics of the 36 patients included in this study are shown in Table 1; the study flowchart is shown in Figure 1. Among the 36 RNU specimens, 33 (92%) were valid for *FGFR3* RNA testing; the remaining three (8.3%) showed test failure due to amplification undetected by reverse-transcriptase-PCR. Although the archival sample age of the three invalid RNU specimens was 5–7 years (failure rate = 20%, three of 15 specimens), no test failure was observed among RNU specimens with an archival sample age of 4 years (Table 1). To investigate whether paired URS biopsy specimens could function as salvage specimens for *FGFR3* RNA testing, paired URS biopsy specimens were obtained from three patients with invalid RNU specimens. All three paired URS biopsy specimens failed *FGFR3* RNA testing. Of the 33 RNU specimens with valid amplification, eight (24%) had *FGFR3*alt as follows: R248C in one, S249C in three, Y373C in three, and FGFR3::TACC3v3 fusion in one. The clinical and molecular details of the eight patients with UTUC harboring RNA-based *FGFR3*alt in RNU specimens are summarized in Table 2. To investigate the concordance of *FGFR3*alt status between RNU specimens and paired URS biopsy specimens, eight URS biopsy specimens were subjected to *FGFR3* RNA testing. Two URS biopsy specimens (25%) were valid for *FGFR3* RNA testing: one (Case ID 8) showed positive concordance with the paired RNU specimen, whereas the other (Case ID 7) did not. The remaining six specimens (75%) showed test failure due to undetected amplification in the URS biopsy specimens. Overall, only two (18.2%) of the 11 URS biopsy specimens were valid for FGFR3 RNA testing.

**Table 1:** Characteristics of 36 patients with upper urinary tract carcinoma undergoing diagnostic URS biopsy and RNU.

**Variables**	**Category**	**Total**	***FGFR3* RNA test of RNU specimen**			
			**Test failure**	**No alteration**	**Alteration**	***P* value #**
No. of patients	Total	36 (100%)	3 (8.3%)	25 (69%)	8 (22%)	-
Treatment period (archival sample age)	2018–2020 (5–7 years)	15 (42%)	3 (100%)	10 (40%)	2 (25%)	0.20 †
	2021–2022 (3–4 years)	7 (19%)	0	5 (20%)	2 (25%)	
	2023–2025 (2 years or less)	14 (39%)	0	10 (40%)	4 (50%)	
Sex	Male	25 (69%)	2 (67%)	16 (64%)	7 (88%)	0.38 †
	Female	11 (31%)	1 (33%)	9 (36%)	1 (13%)	
Age at RNU	(mean ± SD)	73 ± 7.1	71 ± 9.8	74 ± 4.7	69 ± 8.5	0.15 ‡
Tumor grade in URS biopsy specimen	Low-grade	9 (25%)	3 (100%)	4 (16%)	2 (25%)	0.60 †
	High-grade	24 (67%)	0	19 (76%)	5 (63%)	
	Undefined	3 (8.3%)	0	2 (8.0%)	1 (13%)	
H-score of FGFR3 in URS biopsy specimen	Mean ± SD	48 ± 67	1.7 ± 2.8	21 ± 24	150 ± 70	< 0.0001 ‡
	Median, IQR	25 (0–58)	0 (0–5)	15 (0–40)	150 (85–203)	
Tumor grade in RNU specimen	Low-grade	6 (17%)	0	3 (12%)	3 (38%)	0.14 †
	High-grade	30 (83%)	3 (100%)	22 (88%)	5 (63%)	
CIS in RNU specimen	No	29 (81%)	3 (100%)	18 (72%)	8 (100%)	0.15 †
	Yes	7 (19%)	0	7 (28%)	0	
Pathological T (pT) category ##	pTa	6 (17%)	0	3 (12%)	3 (38%)	0.17 †
	pTis	1 (2.8%)	0	1 (4.0%)	0	
	pT1	3 (8.3%)	1 (33%)	1 (4.0%)	1 (13%)	
	pT2	7 (19%)	1 (33%)	4 (16%)	2 (25%)	
	pT3	15 (42%)	0	13 (52%)	2 (25%)	
	pT4	4 (11%)	1 (33%)	3 (12%)	0	
H-score of FGFR3 in RNU specimen	Mean ± SD	112 ± 88	107 ± 100	78 ± 64	218 ± 74	0.0078 ‡
	Median (IQR)	100 (33–178)	100 (10–210)	60 (20–140)	225 (150–293)	

CIS: carcinoma in situ; FGFR3: fibroblast growth factor receptor 3; H-score: histochemical scoring method; IQR, interquartile range; RNU: radical nephroureterectomy; SD, standard deviation; URS: ureteroscopic. #: Compared between no *FGFR3* alteration and *FGFR3* alteration according to *FGFR3* RNA test; ##: non muscle-invasive disease (Ta/is/1) vs muscle-invasive disease (T2-4); †: the Fisher's exact test; ‡: Mann–Whitney U test; Totals may not add up to 100% due to rounding.

**Table 2:** A list of 8 patients harboring *FGFR3* alteration in RNU specimens: comparison between paired URS and RNU specimens.

**Case ID**	**Age (yo)**	**Sex**	**Archival specimen age**	**Diagnostic URS biopsy specimen**			**RNU specimen**		
				**Tumor grade**	***FGFR3* RNA test**	**FGFR3 IHC H-score**	**Pathological T (pT) category and tumor grade**	***FGFR3* RNA test**	**FGFR3 IHC H-score**
1	68	Male	6	High-grade	Test failure	60	pTa low-grade	Y373C	180
2	73	Male	5	Low-grade	Test failure	160	pTa low-grade	Y373C	240
3	55	Male	4	Low-grade	Test failure	100	pT3 high-grade	S249C	100
4	62	Male	4	High-grade	Test failure	210	pTa low-grade	S249C	270
5	59	Male	2	Undefined	Test failure	140	pT2 high-grade	R248C	300
6	69	Male	1	High-grade	Test failure	80	pT2 high-grade	*FGFR3*::TACC3v3	140
7	74	Female	1	High-grade	No alteration	180	pT3 high-grade	Y373C	210
8	81	Male	1	High-grade	S249C	270	pT1 high-grade	S249C	300

FGFR3: fibroblast growth factor receptor 3; H-score: histochemical scoring method; IHC: immunohistochemical; RNU: radical nephroureterectomy; URS: ureteroscopic; yo: years old.

**Figure 2:**
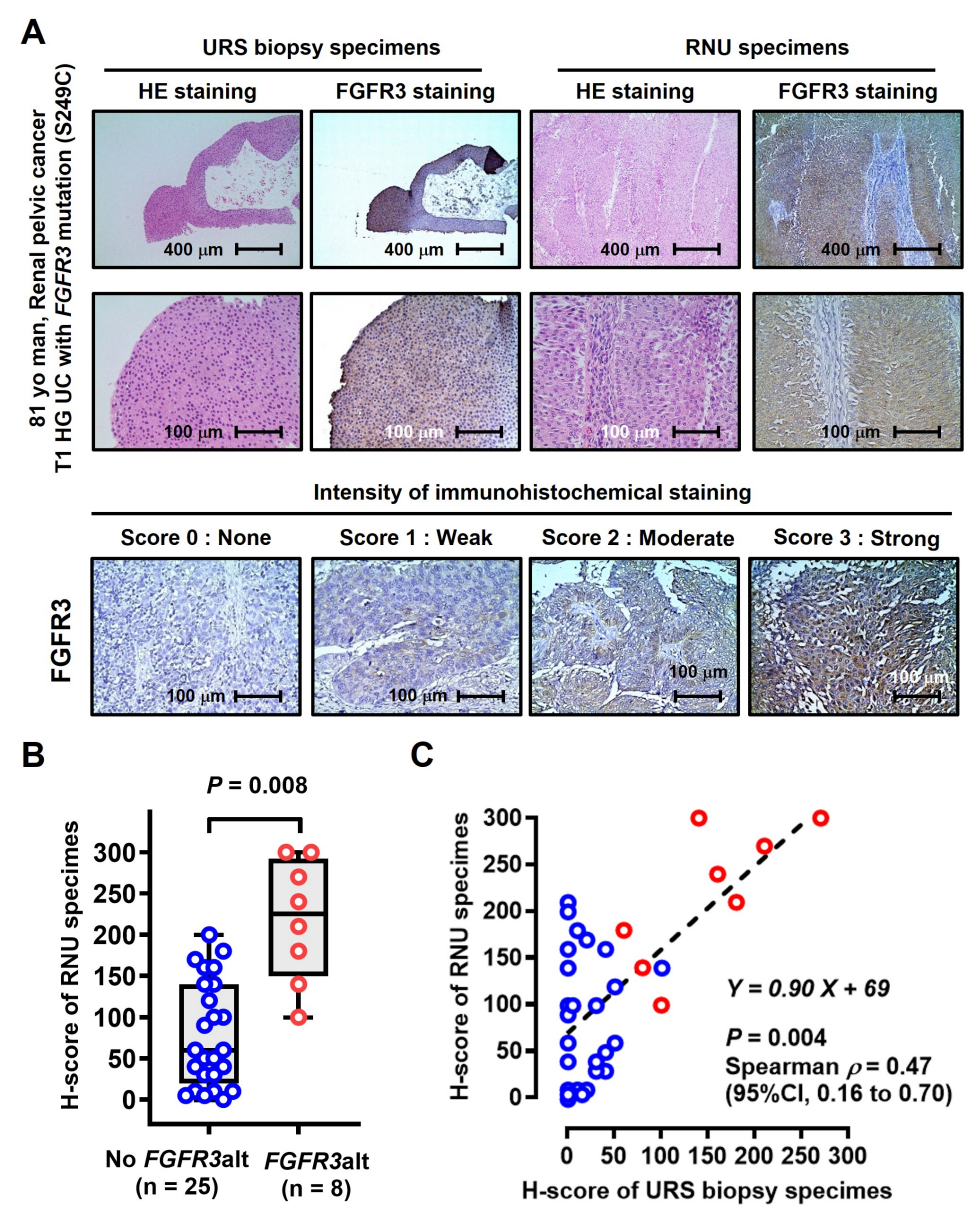
**FGFR3 IHC staining analysis of paired RNU specimens and URS biopsy specimens.** (**A**) FGFR3-IHC images and matched HE-stained images of a representative case (Case ID 8). Representative images showing FGFR3 staining intensity scores of zero to three in upper urinary tract urothelial carcinoma. (**B**) FGFR3 H-scores of RNU specimens are presented using scatter plots and Tukey box-and-whisker plots and compared between patients without *FGFR3*alt and those with *FGFR3*alt. (**C**) Correlation of FGFR3 H-scores between RNU specimens and paired URS biopsy specimens examined using Spearman’s correlation coefficient and linear regression analysis. FGFR: fibroblast growth factor receptor; HE: hematoxylin and eosin; H-score: histochemical scoring method; HG: high-grade; IHC: immunohistochemical; RNU: radical nephroureterectomy; UC: urothelial carcinoma; URS: ureteroscopic; UTUC: upper urinary tract urothelial carcinoma; yo: years old.

We investigated the potential association between RNA-based *FGFR3*alt and IHC-based FGFR3 expression in UTUC. FGFR3-IHC images and matched HE-stained images of a T1 high-grade UC of the renal pelvis with an *FGFR3* S249C mutation (Case ID 8 in Table 2) are shown as a representative example; FGFR3-IHC images with staining intensity scores of zero to three are shown as a reference (Figure 2A). The FGFR3 H-score of *FGFR3*alt-positive RNU specimens was significantly higher than that of *FGFR3*alt-negative RNU specimens (Figure 2B; 218 ± 74 vs. 78 ± 64; *P* = 0.008). Correlation analysis demonstrated a positive correlation between FGFR3 H-scores of RNU specimens and those of the paired URS biopsy specimens (Figure 2C; Spearman’s ρ = 0.47, *P* = 0.004).

## Discussion

Molecular targeted drugs, including ICIs, ADCs, and tyrosine kinase inhibitors, have transformed the treatment landscape for patients with advanced UC. In this context, the identification of prognostic and predictive biomarkers for these therapies is an active area of research. Activation of the *FGFR3* gene in UC tissue is currently established as an actionable biomarker to guide erdafitinib therapy. TUR and radical cystectomy (RC) specimens are generally available for patients with bladder UC, whereas diagnostic URS and RNU specimens are available for patients with UTUC. Metastasectomy and biopsy specimens obtained from metastatic lesions are also available for some patients. The optimal selection of archival specimens for genetic testing should be carefully considered to improve the detection rate of specific gene alterations.

The present study did not aim to redefine the principles of FGFR testing; rather, it provides incremental but practice-informing evidence regarding the suitability of URS biopsy specimens for *FGFR3* biomarker analysis. While ANNAR and other studies have highlighted the importance of tissue quantity, RNA integrity, and archival age, they did not evaluate URS biopsy specimens or examine paired URS/RNU concordance. Our dataset uniquely quantifies RNA failure rates in URS specimens and directly compares FGFR3 IHC expression and RNA-based alterations in paired samples. Although the rate of *FGFR* test failure was reported to be 13.1% (1,103 of 8,396 specimens) in the THOR clinical trials, detailed information on the types of specimens used has not been reported [2, 4]. The ANNAR biomarker study concluded that adequate tumor sample amount, RNA quality, and short archival sample age were vital factors for ensuring valid *FGFR* test results [5]. Pouessel et al. [8] focused on tumor heterogeneity of *FGFR3*alt in invasive bladder cancer by investigating superficial and deep tumor compartments in TUR and RC specimens, as well as paired cancer-positive lymph nodes. *FGFR3*alt was detected in 13 of 34 (38%) T1 tumors, with 100% concordance between superficial and deep compartments, whereas *FGFR3*alt was detected in eight of 27 (30%) ≥ T2 TUR superficial samples, with only four (50%) showing identical mutations in the deeper compartment. *FGFR3*alt was detected in 10 of 201 (5%) cancer-positive lymph nodes, all of which were concordant with the corresponding RC specimen. In our cohort, only 2 of 11 URS biopsy specimens (18.2%) yielded valid *FGFR3* RNA results, and only one specimen showed concordance with the paired RNU sample. These quantified failure rates highlight the practical limitations of URS-derived tissue for RNA-based *FGFR3* testing—an aspect not addressed in ANNAR, which did not report sample collection methods or URS-specific performance.

However, there is a lack of evidence regarding the use of URS biopsy specimens for *FGFR3* RNA testing. In this study, only two (18.2%) of the 11 URS biopsy specimens were valid for *FGFR3* RNA testing; only one patient showed concordance between the RNU specimen and paired URS biopsy specimen. Although RNA yield and quality metrics (e.g., DV200, RIN) were not routinely collected for clinical FGFR testing, our findings implied that URS biopsy specimens are unsuitable for *FGFR3* RNA testing, mainly because of the small amount of RNA obtained. These quantified failure rates highlight the practical limitations of URS-derived tissue for RNA-based *FGFR3* testing—an aspect not addressed in ANNAR, which did not report sample collection methods or URS-specific performance. Technical limitations of URS biopsy specimens for RNA analysis include small tissue volume, low tumor cellularity, RNA degradation during fixation, and sectioning-related loss of material. The three RNU specimens that failed *FGFR3* RNA testing had been archived for 5–7 years. Prolonged FFPE storage is known to cause progressive RNA fragmentation due to cross-linking and oxidation, which reduces the likelihood of successful amplification, particularly for assays requiring longer amplicons. This temporal effect is consistent with the ANNAR study, which demonstrated a marked decline in valid FGFR test results in samples older than 3 years. Potential strategies to mitigate these issues include obtaining multiple biopsies, minimizing cautery, optimizing fixation, and prioritizing larger tissue fragments for molecular testing. Alternative molecular approaches, including the potential advantages and limitations of DNA-based next generation sequencing and the emerging but still investigational role of liquid biopsy for *FGFR3* alterations in UTUC. We sought to determine the suitability of URS biopsy specimens for IHC-based biomarker testing. The FGFR3 H-score of *FGFR3*alt-positive RNU specimens was significantly higher than that of *FGFR3*alt-negative RNU specimens, which is consistent with our previous finding that an *FGFR3* missense point mutation is strongly associated with the overexpression of FGFR3 protein in UC cell lines [9]. A comparison of the FGFR3 H-scores between paired RNU and URS biopsy specimens demonstrated a moderate correlation (Spearman’s ρ = 0.47, *P* = 0.004), suggesting that URS biopsy specimens may serve as protein-based biomarkers when RNU specimens are unavailable; however, the correlation is not strong enough to consider URS IHC a definitive surrogate for RNU tissue. Specifically, RNA is highly susceptible to degradation during fixation and long-term storage, and small biopsy fragments with a high surface-to-volume ratio are particularly vulnerable. In contrast, FGFR3 protein is more stable in FFPE tissue, and IHC requires substantially less material than RNA extraction and amplification. Moreover, IHC does not require intact RNA transcripts and is therefore less affected by partial degradation.

The growing availability of FGFR inhibitors raises the theoretical possibility of initiating targeted therapy prior to RNU in selected patients with low-volume primary tumors of UTUC. Such an approach would require reliable *FGFR* testing on diagnostic URS biopsy specimens or percutaneous biopsy, as RNU tissue would not yet be available. However, our findings indicate that RNA-based *FGFR3* testing is rarely successful in URS samples, with only 2 of 11 specimens (18.2%) yielding valid results. This high failure rate makes pre-RNU FGFR testing based on URS tissue impractical at present.

This study has some limitations. First, the sample size was relatively small; there was potential selection bias because of the retrospective nature of this single-center study. Second, only a single anti-FGFR3 antibody was used in the IHC analysis, which may have affected the staining results. Third, the concordance analysis between paired URS and RNU specimens for *FGFR3* alterations was based on only two evaluable URS samples. Given this extremely limited number, the observed concordance should be interpreted with caution, and no definitive conclusions can be drawn regarding the reliability of URS biopsy specimens for *FGFR3* alteration detection.

Taken together, our findings offer practical guidance for specimen selection in UTUC rather than proposing a transformative change in FGFR testing strategy. URS biopsy specimens appear unsuitable for RNA-based *FGFR3* testing but may still serve as useful material for protein-based biomarker assessment when RNU specimens are unavailable. To the best of our knowledge, this study is the first to investigate the suitability of URS biopsy specimens as treatment-directed biomarkers in patients with UTUC. Future advances in molecular techniques, including improved RNA stabilization, DNA-based next generation sequencing panels, or liquid biopsy approaches, may eventually enable reliable pre-RNU FGFR testing. Further studies are required to develop optimal guidelines for specimen selection, with increasing data supporting their suitability and diagnostic value.

## Abbreviations

ADCs: antibody-drug conjugates
FFPE: formalin-fixed, paraffin-embedded
FGFR: fibroblast growth factor receptor
HE: hematoxylin and eosin
H-score: histochemical scoring method
IHC: immunohistochemical
la/mUC: locally advanced or metastatic urothelial carcinoma
PCR: polymerase chain reaction
RC: radical cystectomy
RNU: radical nephroureterectomy
TUR: transurethral resection
UC: urothelial carcinoma
URS: ureteroscopic
UTUC: upper urinary tract urothelial carcinoma

## Declarations

### Availability of data and materials

The data underlying this article will be shared on reasonable request to the corresponding author.

### Copyright

© The Author(s) 2026.
